# Supplementary material for: Netcombin: An algorithm for constructing optimal phylogenetic network from rooted triplets
Source: PLoS One. 2020 Sep 18;15(9):e0227842. doi: 10.1371/journal.pone.0227842 (PMC7500971; doi:10.1371/journal.pone.0227842)
Supplement: S4 File — (ZIP) [file pone.0227842.s004.zip › SequenceData-Triplet/sequenceData-10-20-30-0-Triplet/length 200, numseq 40, rate 10^-7/2 seq.pdf]

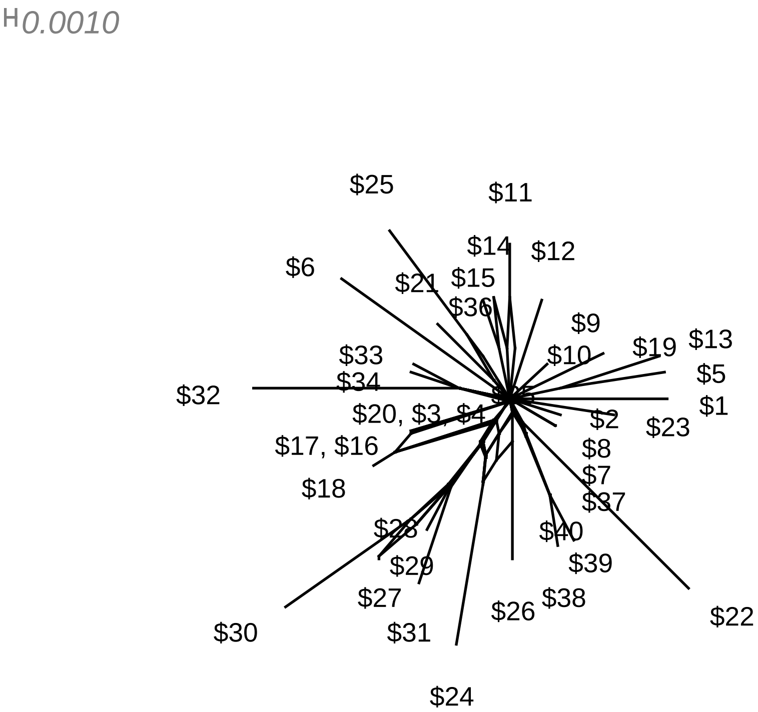

0.0010
$25
$11
$14
$12
$6
$21
$15
$36
$9
$19
$13
$33
$10
$5
$34
$32
$1
$20, $3, $4
$2
$23
$17, $16
$8
$7
$18
$37
$28
$40
$29
$39
$27
$38
$26
$22
$30
$31
$24
